# Possible DNA Viral Factors of Human Breast Cancer

**DOI:** 10.3390/cancers2020498

**Published:** 2010-04-13

**Authors:** Chun-Ru Hsu, Tsong-Ming Lu, Lengsu William Chin, Chi-Chiang Yang

**Affiliations:** 1 Department of Microbiology, National Taiwan University College of Medicine, 1, Jen Ai Road, Sec. 1, Taipei 100, Taiwan; E-Mail: chunruhsu@gmail.com; 2 Institute of Medicine, Chung Shan Medical University, Taichung, 110, Section 1, Chien-Kuo North Road, Taichung 40201, Taiwan; E-Mail: lengsu.chin@msa.hinet.net (L.W.C.); 3 Department of Neurology, Chung Shan Medical University Hospital, 110, Section 1, Chien-Kuo North Road, Taichung 40201, Taiwan; E-Mail: cshy043@csh.org.tw; 4 School of Medical Laboratory and Biotechnology, Chung Shan Medical University, 110, Section 1, Chien-Kuo North Road, Taichung 40201, Taiwan; 5 Department of Clinical Laboratory, Chung Shan Medical University Hospital, 110, Section 1, Chien-Kuo North Road, Taichung 40201, Taiwan

**Keywords:** Epstein–Barr virus, cytomegalovirus, herpes simplex virus, human herpes virus type 8, papillomaviruses

## Abstract

Viruses are considered to be one of the high-risk factors closely related to human breast cancer. However, different studies of viruses in breast cancer present conflicting results and some of these works remain in dispute. DNA viruses, such as specific types of human papillomaviruses (HPV), Epstein–Barr virus (EBV), human cytomegalovirus (HCMV), herpes simplex virus (HSV), and human herpes virus type 8 (HHV-8), have emerged as causal factors of some human cancers. These respective exogenous viruses and the possibility of multiple viral factors are discussed in this review.

## 1. Introduction

Breast cancer is a common malignancy and a major cause of death in women. About 1,150,000 new cases per year are reported worldwide, according to the statistical data of the WHO in 2008. Many epidemiological studies point out a family history of breast cancer is a reproducible predictor of breast cancer risk [1]. Observation of the increased risk of breast cancer for women with positive family histories of breast cancer leads to further sub-classifications into hereditary (familial) or sporadic cases. Some risk factors including obesity have been related to the pathogenesis of breast cancer [2]. Many other factors, including estrogen levels, estrogen receptors, and the adipokines leptin and adiponectin appear to be important in the mechanism of these increased risk factors. Moreover, it was suggested that exogenous and endogenous modulators of oxidative stress may modify the association between the myeloperoxidase polymorphism and breast cancer risk [3]. However, the molecular events in the genesis of this disease are not entirely clear. The reports indicate hereditary breast cancers are responsible for 4–10% of breast carcinomas [4,5,6]. Partially, but importantly, mutations in the BRCA-1, BRCA-2, and P53 genes have been linked to breast carcinomas. Some studies have suggested that the vast majority of multiple-case breast cancer families and families with breast and ovarian cancer would be caused by mutations in the BRCA1 or BRCA2 genes [5,7], but some analyses have suggested these initial studies may have overestimated the prevalence of BRCA1/BRCA2 mutations in hereditary breast cancer cases and mutations in these genes account for about 16% of the familial risk of breast cancer [8,9,10]. The P53 gene mutation is a very frequent event in many human cancers and is associated with a poor clinical outcome in breast cancer patients. In sporadic breast cancers, BRCA1, BRCA-2, and P53 genes mutations may be less common than in hereditary breast cancers. However, our recent results showed no mutation on BRCA-1 and one sample (1.5%) with unreported multiple mutations on BRCA-2 in 69 non-familial breast cancer samples. Fourteen of the 69 samples (20.3%) showed unreported mutations on P53 exons 5-8. The mutation rate of P53 is significantly higher than BRCA-2 and BRCA-1 (unpublished data). Further, viruses have been found in breast cancer tissues. Many widespread chronic diseases, previously thought to be due to metabolic imbalances or genetic modifications, are increasingly being linked to infectious events. Viruses are one of the high-risk factors closely related to human cancers. A viral etiology has been proposed in several human cancers, e.g. EBV in undifferentiated nasopharyngeal carcinoma and Burkitt’s lymphoma [11,12,13], and hepatitis B and C in hepatocellular carcinoma [14]. It is estimated that viral oncogenesis is an etiological factor for 20% of all human cancers [15]. Therefore, the correlation of viruses with breast cancers cannot be excluded. Although it remains to be clarified, the hypothesis a virus may cause human breast cancer has fascinated researchers for decades.

The involvement of viruses in certain breast tumors and cells lines has been described in different studies. Viruses, such as specific types of human papillomaviruses (HPV), Epstein-Barr virus (EBV), and human herpes virus type 8 (HHV-8), have emerged as major causal factors of some human cancers. The presence of either human papillomavirus (HPV)-16 or HPV-18 in the breast has been suggested to be related to the development of a malignant phenotype [16]. Epstein-Barr virus (EBV) was more frequently detected in malignant tissue by polymerase chain reaction (PCR) compared to non-cancerous tissue [17]*.* Late exposure to human cytomegalovirus (HCMV) was reported as a risk factor for breast cancer [18]. An association of human herpesvirus (HHV)-8 with breast cancer has also been suggested [19]. In addition, herpes simplex virus (HSV)-1 DNA was detected in some of the tissues from patients with breast cancer or fibroadenoma [20]. Exogenous retrovirus has also been implicated in the etiology of breast cancer. Table 1 summarizes the results of our studies for the gene mutations/virus presentation in breast cancer. The possible involvement of these viruses in human breast cancer is described separately in the following sections.

**Table 1:** Gene mutations/virus presentation in breast cancer.

Gene/ virus	Sporadic breast cancer	Familial breast-ovarian cancer	Family breast cancer
Gene			
*BRCA1*	0	10–40%	1–11%
*BRCA2*	1.5%	5–15%	5–15%
*P53*	20.3%		
Virus			
HPV	12.9%		
HSV-1	12.9%		
HSV-2	0		
EBV	45.2%		
HCMV	75.8%		
HHV-8	43.8%		

The percentages of virus presentation in non-breast cancer were not shown.

## 2. HPV

Human papillomaviruses belongs to the papillomavirus family, *Papillomaviridae*, capable of infecting humans and the most prevalent sexually transmitted viral infection. It is estimated that 80% of sexually active adults have been infected with at least one HPV type [21]. HPV-16 and HPV-18 are the two most carcinogenic HPV types and are responsible for about 70% of cervical cancer [22]. Our previous study also suggested viral factors, especially HPV, are closely related to cervical cancer [23]. Moreover, in our previous oral cancer study, we demonstrated the possibility of oral cancer caused by virus infection, except for HPV, is very low [24]. 

Although it is well established that high-risk HPVs are the major causal agent for cervical cancer, involvement of the virus in breast cancer is more controversial. The presence of HPV high-risk types 16, 18 and 33 in breast cancer specimens from diverse populations has been demonstrated in previous studies around the World including studies from Italy, Norway, China, Japan, USA, Austria, Brazil, Australia, Taiwan, Turkey, Greece, Korea, Mexico, Hungary and Syria [16,20,25,26,27,28,29,30,31,32,33,34,35,36,37,38], with a positivity variation from 4% to 86% for suggesting negative or positive relationships. 

The positive relationship between HPV and breast cancer was first reported by demonstrating the presence of HPV-16 DNA in 29.4% of 17 breast carcinoma samples [25]. Women positive for both breast and cervical cancers were found to be infected with the same HPV type in both tumors [26,29]. Hennig *et al.* [26] described the presence of HPV-16 in 46% of both ductal and lobular breast carcinomas in women with a history of HPV-16 positive high grade cervical intraepithelial neoplasia (CIN III) lesions. Widschwendter *et al.* [29] found HPV-16 DNA in breast cancer tissues of patients presenting cervical cancer history. Damin *et al.* [16] suggested the presence in the breast of either HPV-16 or -18 might be related to the development of a malignant phenotype. De Villiers *et al.* [30] and Kan *et al.* [31] identified HPV in breast tumors and established HPV as strong candidate oncoviruses for breast cancer. Yasmeen *et al.* [39] proposed HPV-16 is present in many invasive and metastatic breast cancers and less frequently in *in situ* breast cancer. High-risk HPV infection may induce cell invasion and metastasis in breast cancer [39]. Recently, Heng *et al.* [40] further correlated the presence of HPV with the histopathological features of HPV-induced transformation. By *in situ* and standard PCR (with sequencing) and histology-based on light microscopy, the presence of HPV was found in breast cancer cell lines, in the nuclei of cells within the cancerous regions of breast cancer specimens.

However, Gopalkrishna *et al.* [41] suggested the absence of HPV DNA in breast cancer. Recently, Lindel *et al.* [42] also failed to detect HPV DNA by PCR in 81 paraffin-embedded samples of breast cancer. De Cremoux *et al.* [43] reported no HPV-DNA sequences were detected in samples using DNA amplification by PCR. Using PCR and Southern hybridization, our previous study also suggested HPV is not associated with the etiology of breast cancer, although eight (12.9%) out of 62 breast cancer samples were positive for HPV [20]. However, we suggested HPV is significantly related to overall but not relapse-free survivals in breast cancer patients [44]. These results argued against the role of oncogenic HPV in the pathogenesis of breast cancer. The controversy surrounding the role of HPV in breast cancer can be explained by the difficulty that has been encountered in detecting the virus in breast specimens, since a considerable proportion of breast cancer specimens may be non-cancerous and the levels of virus may be low in breast cancer.

## 3. HSV

Herpes simplex virus 1 and 2 (HSV-1 and HSV-2) are two members of the herpes virus family, *Herpesviridae*, that infect humans of all ages. HSV-1 is usually acquired orally during childhood, but may also be sexually transmitted. HSV-2 is primarily a sexually transmitted infection. Human breast cancer cells are permissive to HSV [45]. HSV is highly infectious and can be used both as a delivery vector for cancer therapeutic genes and as a backbone for oncolytic virus [46,47]. The inherently direct cytocidal effect of HSV, if harnessed and made to be selective by genetic manipulations, makes this virus a good candidate for developing viral-based oncolytic approaches [48]. A neurovirulent gene-deleted HSV strain has recently been used to target breast cancer cells for the treatment of breast cancer [49]. However, our previous findings demonstrated the human cancer cells, KB, which contain HPV 18 sequences can be infected by HSV-1, the viability was increased and the DNA fragmentation was decreased [50]. This suggested that KB cells can survive HSV-1 infection. The safety of using HSV as viral vector for gene therapy thus should be re-considered.

As for the oncogenic potential, our previous study suggested that HSV-1 could be a cofactor in the oncogenesis of oral cancer [24]. Utilizing PCR and Southern hybridization to detect virus DNA in patients with breast cancer and specimens from non-cancerous or other individuals with thyroid tumors or fibroadenoma, we reported HSV-1 is associated with benign (fibroadenoma), but not malignant tumors of breast tissue [20]. Comparing the fibroadenoma group with the non-cancerous group, the results showed HSV-1 was closely related to fibroadenomas, which are the most common benign tumors of the female breast and are associated with a slight increase in the risk of subsequent breast cancer [51]. The study by Hildesheim *et al.* [52] suggested HSV-2 increased the rate of development of cervical neoplasia. However, our previous studies suggested HSV-2 is a bystander for cervical cancer and is not determinative for oral cancer [23,24]. Both types of HSV might not be involved directly in the oncogenic processes. So far, there is no direct evidence for association between HSV and breast carcinogenesis. Whether HSV is involved in breast cancer is not conclusive, although HSV-1 was demonstrated to relate to the overall survival of breast cancer patients [44]. 

## 4. EBV

The EBV is a cancer-causing virus of the herpes family, *Herpesviridae*, including herpes simplex virus 1 and 2, and is one of the most common viruses in humans worldwide. Most of the world’s population tolerates lifelong EBV infection with no adverse health consequences under the balance of EBV persistence, virion production, and immune control. However, EBV has been linked to the etiology of several cancers, including African Burkitt’s lymphoma [11]; Hodgkin, AIDS, and nasal NK/T-cell lymphomas; post-transplant lymphoproliferative disorder; nasopharyngeal carcinoma (NPC); lymphoepithelioma-like squamous cell malignancies; gastric adenocarcinoma; and leiomyo-sarcoma [12,13]. The association of EBV with breast cancers has been proposed, but findings from investigations of this association have been widely variable.

The presence and expression of EBV in breast tumor subpopulations (up to 50%, but frequently less) has been demonstrated in several studies [17,20,53,54,55,56,57]. Horiuchi *et al.*, [58] reported two out of three breast carcinomas were positive for EBV by PCR, but upon *in situ* hybridization, the EBV was localized in infiltrating lymphocytes, not the breast epithelium. Labrecque *et al.* [17] detected EBV by PCR in 21% of a series of 91 breast cancers. By PCR and Southern hybridization, our previous study [20] showed EBV was significantly associated with non-familial breast cancer (P < 0.01). Trabelsi *et al.* [59] observed positivity of EBV in tumor cells, but not in non-tumoral epithelial cells nor in lymphoid cells, suggesting possible involvement of EBV in these tumors. By immunohistochemistry, Joshi *et al.* [60] suggested EBV nuclear antigen-1 (EBNA-1) expression is seen in a significant proportion of breast cancer tissue specimens, as compared to patients with benign breast diseases. By both immunohistochemistry and PCR, Fawzy *et al*. [61] also suggested EBV is significantly associated with breast cancer. Lin *et al.* [62] established EBV infected breast cancer MCF7 and BT474 cells and demonstrated EBV infection promotes tumorigenic activity of breast cancer cells. Breast epithelial cells can be infected by EBV through direct contact with EBV-bearing lymphoblastoid cells and EBV infection has been shown to confer increased resistance to chemotherapeutic drugs in breast cancer cells [62]. It is suggested entry of EBV into epithelial cells involves an epithelial-specific receptor [63,64] or cell–cell contact with infected lymphocytes [65]. 

However, some earlier negative results for EBV as the etiology of breast cancer have also been reported. Three other groups in the United States, Belgium, and Taiwan failed to find evidence of EBV by PCR in medullary breast carcinomas [66,67,68] or invasive ductal carcinomas [68,69]. Moreover, some researchers found EBV gene products or genomic materials are absent in breast cancer cells [69,70,71,72]. Moreover, a low viral load was detected in breast cancer biopsy specimens and infected cells were not clearly identified in most of the available studies. Micro-dissection and isolation of pure tumor cells have found even in EBV-positive tumor samples, many tumor cells do not contain EBV genomes and breast carcinomas are highly heterogeneous in terms of genome content and distribution [73]. Such findings raise the possibility that although EBV might not to present an etiological role in the genesis of breast cancer, the virus might contribute to tumor progression. If so, it is interesting for our previous results to suggest that EBV is related to overall survival, but not relapse-free survival of breast cancer [44].

## 5. HCMV

HCMV is a β-herpesvirus of *Herpesviridae* causing widespread, persistent human infections. HCMV spreads mainly by the sexual and transfusion routes and an association with cervical cancer has been suggested [74]. Based on our previous studies, it may be suggested HCMV is a cofactor in the oncogenesis of cervical cancer, however, is not determinative and might not be directly associated in the oncogenic processes of oral cancer [23,24]. Moreover, as revealed by our previous study, HCMV was the only virus related to thyroid tumor and the presence of HCMV in mammary tissues may be common and might occur less frequently in thyroid tissues than in breast tissues [20]. Further, late exposure (in adulthood) to a common virus, such as human cytomegalovirus, has been hypothesized as a risk factor for breast carcinomas [18]. Breathnach *et al.* [75] reported a unique case of HCMV pneumonitis in a woman with stage IV breast cancer, receiving both chemotherapy and systemic corticosteroids. Richardson *et al.* [76] further provided evidence of an association between HCMV IgG levels and breast cancer in young women. By measuring HCMV IgG levels in stored plasma from 208 women with breast cancer and 169 controls, higher mean HCMV IgG levels were found in women with breast cancer. This could be the result of a more recent infection with HCMV, and potentially confirms that late exposure to HCMV is a risk factor for breast cancer. Interestingly, it is consistent with our previous study to suggest HCMV as well as human herpesvirus-8 are the only two studied DNA tumor viruses closely related to relapse-free and overall survivals of breast cancer [44]. It is suggested HCMV could be associated with breast cancer because it is a ubiquitous virus that is shed in breast milk, as well as in saliva, urine, cervical secretions, and semen, impling HCMV persistently infects epithelial cells [76,77]. However, whether the association between HCMV and breast cancer is causal requires further investigation. 

## 6. HHV-8

HHV-8, also known as Kaposi’s sarcoma-associated herpesvirus (KSHV), is the eighth human virus of the *Herpesviridae*. This virus causes Kaposi's sarcoma, a cancer commonly occurring in AIDS patients, as well as primary effusion lymphoma and some types of multicentric Castleman's disease. The role of this virus in causing cancer has been well-established for over a decade [78]. Our previous study suggested HHV-8 was closely related to fibroadenomas [20], however, it was not related to oral and cervical cancers [23,24]. Several recent studies have described HHV-8 as being detected in breast cancer patients. Newton *et al.* [19] suggested HHV-8 was related to breast cancer by the immuno-serological method. By PCR and Southern hybridization, we also reported that HHV-8 was the most significantly associated virus with breast cancer and occurred more frequently than EBV [20]. Our further study demonstrated HHV-8 was related to human breast cancer in both overall and relapse-free survivals [44]. 

HHV-8 is linked to a number of malignancies thought to be driven by cytokines, including interleukin-6 (IL-6). Higher levels of IL-6 are relevant to many cancers such as prostate cancer [79], ovarian cancer [80], and indolent lymphoma [81]. Moreover, recent reports pointed out serum IL-6 levels correlate to tumor progression and prognosis in metastatic breast carcinoma [82,83,84,85]. HHV-8 has unique genes ORF K2 and K14 which encode viral IL-6 (vIL-6) and neural cell adhesion-like protein (OX-2), respectively. The vIL-6 is a homolog of human IL-6 (hIL-6) and it does not require the nonsignaling alpha-receptor subunit for the formation of CD130-based signal transducing complexes [86,87,88], whereas OX-2 up-regulates Th2 cytokines and increase inflammatory cytokines such as IL-6 production [89]. Activation of the hIL-6 gene by Rta, a transcriptional activator encoded by HHV-8, activates the viral lytic cycle leading to the expression of several viral genes implicated in viral pathogenesis supports the role of hIL-6 in the development of malignancies [90]. It was suggested that there operates a combination of cell transformation mediated by latently expressed proteins that promote cell proliferation and survival coupled with paracrine signaling functions mediated by either the viral cytokines or viral receptor-induced secreted cellular proteins [91]. IL-6 induces increased motility, cell-cell and cell-substrate dyshesion and epithelial-to-mesenchymal transformation in breast cancer cells [92]. It was reported the functional polymorphism −174G > C within the IL-6 gene promoter is not associated with breast cancer risk [93]. However, IL-6 was found to increase expression and secretion of cathepsin B by breast tumor-associated monocytes [94]. Our preliminary data also suggested significant increased IL-6 in breast cancer patients compared with hepatitis cancer patients and healthy individuals (P < 0.01) (unpublished data). These results imply the close association of HHV-8 with breast cancer. However, whether HHV-8 plays a causal role in breast cancer remains unclear. 

## 7. A Possibility of Multiple Viral Factors Involved in Breast Cancer

In our previous report we suggested that HHV-8 is more frequently associated with breast cancer than EBV and the number of infecting viruses associated with benign breast fibroadenoma was higher than for malignant breast cancer [20]. Moreover, HSV-1 is associated with benign (fibroadenoma) but not malignant tumors (breast cancer) of breast tissue. The findings of our earlier studies with respect to the significance of viral factor to the overall survival, relapse-free survival and oncogenic potential/associated virus of breast cancer, fibroadenoma, thyroid tumor, cervical cancer, and oral cancer patients are summarized in Table 2 [20,23,24,44,95]. Different roles of each virus for different cancers were postulated. Based on these results, it seems reasonable to suggest HSV-1, HHV-8, EBV, HCMV and HPV are related to overall survival, however, only HHV-8 and HCMV appear related to relapse-free survival of breast cancer. It is thus reasonable to speculate more virus infection by more virus types, *i.e.*, multiple virus infection, would have greater effects on the immune system as well as overall and relapse-free survivals. In our previous report, we suggested the HHV-8 virus had the strongest association with breast cancer tissues [20]. This finding was reinforced with statistically significant relationships demonstrated between HHV-8 and both overall and relapse-free survivals. Thus, we suggested the importance of HHV-8 with respect to the oncogenic processes and survival of breast cancer cannot be ignored. For HCMV, it was suggested it is related to the rate of relapse-free and overall survivals [44] but not oncogenesis in breast cancer [20]. Recently, Cengiz *et al.* [96] have shown statistically significant increases in the number of metastatic lymph-nodes, vascular invasion and tumor size for breast cancer patients with thyroid disease and greater frequency of thyroid pathology compared to normal controls. Interestingly, we have previously suggested HCMV is the only virus associated with thyroid tumors [20]. If proven, this raises the possibility that it could explain the increased numbers of metastatic lymph-nodes and confirms that late exposure to HCMV is a risk factor for breast cancer as described above. For EBV, despite the associated oncogenic potential of EBV with breast cancer [20], a relationship with relapse-free survival has not been proposed. Widschwendter *et al.* [29] suggested HPV may be transported from the original site of infection (cervical tissues) to the breast through the blood stream, and this may be the reason for the detection of HPV DNA in breast cancer samples in which there is a cervical cancer history. This is consistent with our previous findings HPV is significantly related to cervical and oral cancers but not to breast benign or malignancy and thyroid tumor [20,23,24,44,95]. Moreover, it has been demonstrated HPV is significantly related to overall but not relapse-free survival in breast cancer patients [44]. 

Based on parts of the DNA oncogenic viruses studied, for the association of virus with breast cancer, multiple virus infection is significantly related to the overall and relapse-free survivals of breast cancer patients. This is consistent with our previous finding that the presence of multiple viruses predominates in breast cancer [20]. If this is true, immune-suppressive chemotherapy may further influence the virus infectious status. Moreover, the overall and relapse-free survivals of the multiple (more than two) virus-infected breast cancer patients group is significantly differs from the no virus- or one virus-infected group [44]. Therefore, we suggest multiple viral factors may be related to human breast cancer not only in terms of the oncogenic processes revealed in our previous tissue-specimen study, but also in terms of overall and relapse-free survival, ultimately reflecting treatment efficacy.

**Table 2:** Summary of the significance of viral factor to the overall survival, relapse-free survival and oncogenic potential/associated virus of breast cancer, fibroadenoma, thyroid tumor, cervical cancer, and oral cancer patients.＋: significant; －: not significant. N.A.: not available. The Arabic number represents the order of significance.

Virus	Breast cancer		Oncogenic potential/associated virus				
	Overall survival	Relapse-free survival	Breast cancer	Fibroadenoma	Thyroid tumor	Cervical cancer	Oral cancer
HPV	＋	－	－	－	－	＋^1^	＋^1^
HSV-1	＋	－	－	＋^1^	－	－	＋^2^
HSV-2	N.A.	N.A.	－	－	－	＋^3^	－
EBV	＋	－	＋^2^	＋^2^	－	－	－
HCMV	＋	＋	－	－	＋	＋^2^	－
HHV-8	＋	＋	＋^1^	＋^1^	－	－	－

The viruses associated with breast cancer are HHV-8 > EBV (P < 0.01). The viruses associated with fibroadenoma were HSV-1 and HHV-8 > EBV (P < 0.01).

## 8. Conclusions

Breast cancer is one of the most frequently diagnosed malignancies of women in many populations. Previous studies provide evidence that viruses exist in patients with breast cancer and suggest viruses can be one of the risk factors for breast cancer [97,98]. Several candidate viruses such as the specific types of HPV, EBV, HCMV, HHV-8, MMTV and HERV have been proposed. However, some of these studies are disputed.

The identification of a MMTV strengthens the possibility of the viral etiology for breast tumors in animals. Although it has been assumed MMTV might be involved in the pathogenesis of human breast cancer, similar viral sequences found in humans cannot provide direct evidence of carcinogenesis. The presence of high-risk HPV, EBV and HHV-8 has been detected in breast cancer specimens, but the association of these candidate viruses with breast cancer has shown inconsistent results in different studies. A more specific approach, such as combining real-time quantitative PCR (to measure the amount of viral load in archival tissue samples) with laser capture microdissection (to improve localization of viral nucleic acid in benign or malignant components of a tissue sample) is suggested for further investigation [73].

Moreover, the possibility of multiple viral factors being involved in breast cancer has also been reported [20,44]. By PCR and Southern hybridization analysis of HSV-1, HPV, HCMV, EBV, and HHV-8 DNA in some of the tissues of patients with breast cancer and non-breast cancer controls, it was found the presence of multiple viruses was not detected in the non-cancerous and thyroid tumor groups, but was common in the breast cancer and fibroadenoma groups [20]. The presence of more than one virus was found predominantly in breast cancer, suggesting multiple viral factors may be associated with breast cancer. If an association between one, or more, of these viruses and breast cancer is verified, it may help develop new anti-viral therapies and vaccine strategies for combating this increasingly common cancer. More efforts are needed to provide conclusive evidence linking viruses to the etiology of breast cancer.
